# Characterization of a New *Saccharomyces cerevisiae* Isolated From Hibiscus Flower and Its Mutant With L-Leucine Accumulation for Awamori Brewing

**DOI:** 10.3389/fgene.2019.00490

**Published:** 2019-05-28

**Authors:** Takayuki Abe, Yoichi Toyokawa, Yukiko Sugimoto, Haruna Azuma, Keiko Tsukahara, Ryo Nasuno, Daisuke Watanabe, Masatoshi Tsukahara, Hiroshi Takagi

**Affiliations:** ^1^BioJet Co., Ltd., Okinawa, Japan; ^2^Division of Biological Science, Graduate School of Science and Technology, Nara Institute of Science and Technology, Ikoma, Japan

**Keywords:** comparative genomics, hibiscus yeast, *Saccharomyces cerevisiae*, breeding, awamori, liquor flavors

## Abstract

Since flavors of alcoholic beverages produced in fermentation process are affected mainly by yeast metabolism, the isolation and breeding of yeasts have contributed to the alcoholic beverage industry. To produce awamori, a traditional spirit (distilled alcoholic beverage) with unique flavors made from steamed rice in Okinawa, Japan, it is necessary to optimize yeast strains for a diversity of tastes and flavors with established qualities. Two categories of flavors are characteristic of awamori; initial scented fruity flavors and sweet flavors that arise with aging. Here we isolated a novel strain of *Saccharomyces cerevisiae* from hibiscus flowers in Okinawa, HC02-5-2, that produces high levels of alcohol. The whole-genome information revealed that strain HC02-5-2 is contiguous to wine yeast strains in a phylogenic tree. This strain also exhibited a high productivity of 4-vinyl guaiacol (4-VG), which is a precursor of vanillin known as a key flavor of aged awamori. Although conventional awamori yeast strain 101-18, which possesses the *FDC1* pseudogene does not produce 4-VG, strain HC02-5-2, which has the intact *PAD1* and *FDC1* genes, has an advantage for use in a novel kind of awamori. To increase the contents of initial scented fruity flavors, such as isoamyl alcohol and isoamyl acetate, we attempted to breed strain HC02-5-2 targeting the L-leucine synthetic pathway by conventional mutagenesis. In mutant strain T25 with L-leucine accumulation, we found a hetero allelic mutation in the *LEU4* gene encoding the Gly516Ser variant α-isopropylmalate synthase (IPMS). IPMS activity of the Gly516Ser variant was less sensitive to feedback inhibition by L-leucine, leading to intracellular L-leucine accumulation. In a laboratory-scale test, awamori brewed with strain T25 showed higher concentrations of isoamyl alcohol and isoamyl acetate than that brewed with strain HC02-5-2. Such a combinatorial approach to yeast isolation, with whole-genome analysis and metabolism-focused breeding, has the potentials to vary the quality of alcoholic beverages.

## Introduction

Awamori, a distilled alcoholic beverage made from steamed rice, is brewed primarily in Okinawa, Japan. This traditional spirit, with more than 600 years of history, is representative of Okinawan culture and industry. Two microorganism species, the fungus *Aspergillus luchuensis* and the yeast *Saccharomyces cerevisiae* play essential roles in awamori brewing. *A. luchuensis*, as Kuro-koji mold, saccharifies steamed rice, and subsequently *S. cerevisiae* produces ethanol by multiple parallel fermentations. After 2 weeks of fermentation, the fermented mash (*moromi*), containing ethanol at a final concentration of approximately 20% (v/v), is obtained and applied to the distillation process to develop a rich and strong flavor. Awamori is composed of various aromatic compounds, which are distinguishable from other types of Japanese spirits (Nishiya, 1980). Two categories of flavors have been well studied in awamori: the initial volatile compounds commonly known as the “top notes” of flavors and the flavors that develop during the aging process (Koseki et al., 1996; Taira et al., 2012). These fruity and sweet flavors are favorable and demanded by consumers. For the awamori industry, it is important to enhance these two categories of flavors.

The initial volatile compounds, including fruity aroma esters and alcohols are recognized as important flavors in awamori (Taira et al., 2012). In particular, the biosynthetic pathway of isoamyl alcohol and its acetate, isoamyl acetate, is elucidated as follows. These metabolites are synthesized from α-ketoisocaproate (KIC), a precursor of L-leucine, in *S. cerevisiae*. Isoamyl alcohol is synthesized from KIC catalyzed by α-keto acid decarboxylase and alcohol dehydrogenase, and is finally converted into isoamyl acetate by alcohol acetyltransferase. In the L-leucine synthetic pathway, α-isopropylmalate synthase (IPMS; the *LEU4* gene product) (EC 4.1.3.12) is the key enzyme, because its activity is regulated by feedback inhibition by L-leucine (Baichwal et al., 1983). Previously, we isolated a mutant of the diploid awamori yeast strain 101-18, which accumulated a higher amount of L-leucine selected from the L-leucine analog 5,5,5-trifluoro-DL-leucine (TFL)-resistant colonies. The mutant strain 101-T55 has mutations in the *LEU4* gene, which confers overproduction of isoamyl alcohol and isoamyl acetate (Takagi et al., 2015). Such yeast breeding technology targeted to specific flavors is applicable to the improvement of the quality of awamori.

Vanillin, which is regarded as a representative flavor of aged awamori, gives a sweet vanilla scent (Koseki et al., 1996). Vanillin is converted from 4-vinyl guaiacol (4-VG), which is produced mainly by koji mold assimilating ferulic acid during awamori fermentation (Koseki et al., 1998; Maeda et al., 2018). In *S. cerevisiae*, the *PAD1* and *FDC1* genes play essential roles in catalyzing the decarboxylation of ferulic acid (Mukai et al., 2010). However, awamori yeast strain 101-18 has the *FDC1* pseudogene with a nonsense mutation, leading to the loss of 4-VG-producing activity (Mukai et al., 2014). Therefore, the isolation or construction of a yeast strain with a functional ferulic acid decarboxylase is anticipated in order to brew novel awamori.

Our objective is to develop a new kind of awamori characterized by the top notes of flavors and altered flavors during aging. It is considered that yeast metabolism greatly influences the contents of isoamyl acetate and vanillin, known as the key flavor compounds in awamori. For awamori brewing, an awamori yeast strain of *S. cerevisiae*, Awamori 101, which is a diploid prototroph (supplied by National Research Institute of Brewing, Japan), has been dominantly used as a standard and conventional strain among awamori breweries. Strain 101-18 with high alcohol productivity has been isolated from Awamori 101 and is applicable for genetic analysis and awamori fermentation test (Takagi et al., 2015). Recently, several types of awamori have been manufactured and commercialized by awamori brewery, which brewed by use of yeast strains derived and isolated from nature in Okinawa, such as mango and brown sugar lump. These unique awamori possess the different characteristics in taste and flavor compared to those prepared by conventional yeast strain. However, there has been little study done concerning the genetic analysis of newly isolated yeast strains. Therefore, it is important to clarify the genetic differences between the novel yeast strain and the other conventional yeast strains. In this study, we aimed to isolate and characterize a new yeast strain that has the potential to promote the production of these compounds in awamori brewing. To achieve this, we applied combinatorial methods for the isolation and breeding of a new yeast strain. We also presented the whole-genome information on yeast strains for awamori to uncover the molecular basis of its fermentation properties.

## Materials and Methods

### Culture Media

*S. cerevisiae* cells were cultured in a nutrient rich medium YPD (2% glucose, 1% yeast extract, 2% peptone). A synthetic defined medium SD+Am [2% glucose, 0.5% ammonium sulfate, 0.67% yeast nitrogen base without ammonium sulfate and amino acids (Difco Laboratories)] was used for analysis of intracellular amino acid contents in yeast. A SD medium containing 0.5% allantoin as a sole nitrogen source (SD+Alt) was used for selection of HC02-5-2 mutants resistant to TFL.

### Isolation of Yeast Strains for Awamori Brewing From Hibiscus Flowers

Wild hibiscus flowers (totally 23) were collected in Okinawa, Japan. The flowers were incubated in YPD medium containing 4% ethanol at 32°C. The resultant muddy media suspension was then incubated on YPD agar plates at 32°C. After 24–48 h incubation, 5 of well-grown single colonies were picked and subsequently applied to a laboratory-scale fermentation test to select yeast strains with high ethanol productivity. The genomic DNA was extracted from yeast cells that produced high concentrations of ethanol by a laboratory-scale fermentation test. PCR was performed to amplify a part of 26S rDNA region (560 bp). PCR was conducted with the following primers: NL1 (5′-GCA TAT CAA TAA GCG GAG GAA AAG-3′) and NL4 (5′-GGT CCG TGT TTC AAG ACG G-3′). The amplified product was confirmed with DNA sequencing and queried the BLAST search program to identify yeast species.

### Awamori Fermentation Test and Distillation

The method for laboratory fermentation test was described previously (Takagi et al., 2015). In brief, 50 g of rice koji, 65 ml of water, and 0.1 ml of precultured yeast cells were mixed in a 200 ml Erlenmeyer flask. The fermented mash was incubated at 25°C for 14 days to prepare final fermented mash (*moromi*). After filtration of the supernatant of *moromi*, ethanol concentrations were analyzed using portable alcohol detector (AL-3; Riken Keiki). To analyze the production of flavor, the distillation of *moromi* was performed by atmospheric distillation using a water distiller (MH943SBS; Megahome) and stopped when the ethanol concentration reached 10%. The distillate was collected and subjected to liquid chromatography and gas chromatography according to the below methods.

### Liquid and Gas Chromatography Analysis

Quantification of 4-VG was performed using Shimadzu HPLC system. Distilled awamori liquids were passed with a 0.45 μm-pore filter. The filtrated samples were loaded on the C18 column and eluted at a flow rate of 0.5 ml/min with an acetic buffer containing methanol. The elution was started with solvent A (50 mM acetic acid, pH4) and gradually increasing solvent B (100% methanol) up to 90%. On chromatogram at 254 nm of absorbance, the peak of 4-VG was detected at the same retention time as the standard solution. The peak area was calculated and the concentrations were determined by comparing to the standard solution.

The volatile compounds were analyzed using the electric nose of Heracles II system (Alpha MOS). The system composed of the solid phase absorption of vapor from samples, the dual gas chromatography columns and the FID detection. The distilled liquids from the laboratory scale fermentation were diluted with distilled water and prepared in 15% alcohol. Each 10 ml sample in glass vials was set on the equipment. The analysis was performed according to the manufacturer’s installation instruction. The volatile compounds were quantified by comparing the peak areas with the standards.

### Genome Sequencing and Phylogenic Analysis

The extracted genome DNA from wild-type strain HC02-5-2 isolated from hibiscus flower, its mutant strain T25, and Japanese sake strain Kyokai no.7 (K7) underwent quantification by Qubit (Thermo). Next generation sequencing library was constructed for each genome using the Nextera DNA Library Preparation Kit (Illumina) according to the manufacturer’s instructions. The genome libraries were sequenced using MiSeq (Illumina) with MiSeq Reagent Kit v2 or v3 (Illumina). Sequencing data processing of strains HC02-5-2, T25, and K7, as well as sequencing data from the Sequence Read Archive (SRA) was performed with CLC Genomics Workbench v 10.1.1 (QIAGEN). This included trimming, mapping, and variants calling against the reference genome of *S. cerevisiae* S288c (GCA_000146045). Reads bases not matching in the alignment were scored as variants. The coverage table files and the variants table files were exported from Genomics Workbench and retained for further analysis. These files were converted to a fasta file of synthetic sequences with custom scripts^1^. These scripts generate the sequences of homozygous SNPs from the data of coverage and variants. The fasta file was applied to the phylogenetic analysis with the Neighbor-joining method using MEGA X: Molecular Evolutionary Genetics Analysis software (Kumar et al., 2018). Parameters: Statistical method; Neighbor-joining, Substitution model; Maximum composite likelihood, Substitutions to include; d: transitions + transversions, Rates among sites; uniform rates, pattern among lineages same (homogeneous). SRA accession numbers are shown in Supplementary Table 1. The parent strain HC02-5-2 has 10,569 homozygous and 388 heterozygous variants to the reference genome of *S. cerevisiae* S288c. The mutant strain T25 has 10,763 homozygous and 483 heterozygous variants to the S288c reference genome. The comparison of T25 to its parental strain HC02-5-2 revealed 287 homozygous and 231 heterozygote specific variant sites.

### Isolation of TFL-Resistant Yeast Mutant

To induce mutations, HC02-5-2 cells were treated with 6% ethyl methanesulfonate (EMS) (Rose and Broach, 1991). The mutagenized cells were spread onto SD+Alt agar plates containing 40 μg/ml of TFL and incubated at 30°C for 3 days. Resultant colonies were cultured in SD liquid medium and subsequently subjected to an amino acid analyzer for selecting the L-leucine-accumulating mutants.

### Gene Cloning and Plasmid Construction

The centromere-based low-copy-number plasmid pYC130 containing the G418 resistance gene (KanMX4) (supplied by National Research Institute of Brewing, Hiroshima, Japan) (Calera et al., 2000) and the 2 μ-based high-copy-number plasmid pAD4 containing the *ADH1* promoter and terminator (supplied by J. Nikawa, Kyushu Institute of Technology, Fukuoka, Japan) (Nikawa et al., 2006) were used to subclone and express the *LEU4* gene, respectively. *Escherichia coli* strain DH5α [F^-^λ^-^Φ*80lacZΔM15 Δ(lacZYA argF)U169 deoR recA1 endA1 hsdR17*(*r*_k_^+^*m*_k_^-^) *supE44 thi-1 gyrA96*] was used to construct plasmids.

Full-length *LEU4* gene was amplified from the genomic DNA of HC02-5-2 cells by using KOD FX Neo polymerase (Toyobo), with addition of the *Hin*dIII and *Sac*I recognition site at 5′ and 3′ ends of the coding region of *LEU4*, respectively. PCR products were cloned into *Hin*dIII – *Sac*I site in pAD4 (pAD4-LEU4) and then PADH1-LEU4-TADH1 fusion was amplified from pAD4-LEU4, with addition of the *Kpn*I and *Mlu*I recognition site at 5′ and 3′ ends, respectively, at the region of PADH1-LEU4-TADH1 fusion. Amplified products were subcloned into pYC130 to construct expression plasmid (pYC130-LEU4) in yeast cells. Plasmids for expressing the Leu4 variants [pYC130-LEU4(G516S), pYC130-LEU4(S452F/A551V) and pYC130-LEU4(G516S/S542F/A551V)] were prepared using Quick-Change II Mutagenesis Kit (Agilent Technologies). Site-specific mutagenesis for constructing plasmids were performed with the following primers: *LEU4* (G516S) q-change Fw (5′-GAA GGT ACA GGT AAT AGT CCA ATC TCT-3′), *LEU4* (G516S) q-change Rv (5′-AGA AGA GAT TGG ACT ATT ACC TGT ACC TTC-3′), *LEU4* (S542F/A551V) q-change Fw (5′-TCT CGT AGC AAA CTA CAC AGA GCA TTT TCT AGG TTC TGG TTC TTC TAC GCA AGT TGC TTC TTA CAT CCA TC-3′), *LEU4* (S542F/A551V) q-change Rv (5′-GAT GGA TGT AAG AAG CAA CTT GCG TAG AAG AAC CAG AAC CTA GAA AAT GCT CTG TGT AGT TTG CTA CG-3′). Expression plasmids were transformed into HC02-5-2 cells using the lithium acetate method (Rose and Broach, 1991) and transformants were selected on YPD agar plates containing 200 μg/ml of G418. The DNA sequences of the amplified products with PCR and plasmids newly constructed in this study were confirmed by sequence analyses based on the Sanger method by using ABI PRISM 3130 Genetic Analyzer (Applied Biosystems).

### α-Isopropylmalate Synthase (IPMS) Activity Assay

Yeast cells were cultured on YPD liquid medium at 30°C for 2 days. Culture medium was centrifugated for collecting yeast cells and the pellet was suspended in 250 mM Tris-HCl (pH 8.5) containing 1 mM phenylmethylsulfonyl fluoride (PMSF). Cell suspension was treated with a Multi-Beads Shocker (Yasui Kikai) at 4°C, for preparing the whole-cell extracts by disrupting with glass beads, and resultant supernatant was used for crude enzyme solution. IPMS activity was measured as described previously (Takagi et al., 2015). One unit of enzyme activity is defined as the amount of coenzyme A (CoA) liberated from acetyl-CoA, which produced by an enzymatic transacylation reaction from 2-ketoisovalerate to 2-isopropylmalate, at 37°C for 60 min. Protein concentration was measured using a Bio-Rad Protein Assay Dye reagent concentrate, which employs the Bradford method. The protein concentrations of each sample were estimated from the index of absorbance at 595 nm on the basis of a standard curve of bovine serum albumin.

### Quantification of Intracellular Amino Acids Contents

Yeast cells were cultured in 5 ml of SD+Am medium containing 200 μg/ml of G418 (if necessary) at 30°C for 2 days (OD_600_ = 10.0). Collected cells were suspended in 500 μl of distilled water and boiled at 100°C for 20 min to extract intercellular amino acids. After centrifugation at 13,000 × *g* for 5 min, supernatant was filtered with 0.2 μm syringe filter (mdiTM). Filtrated samples were subjected to an amino acid analyzer (AminoTacTM JLC-500/V, JEOL) or a LC/MS amino acid system (UF-Amino Station, Shimadzu) for quantifying amino acids contents in yeast cells. Experimental procedures for analyzing amino acid content by LC/MS were conducted as reported previously (Shimbo et al., 2009). The content of each amino acid was expressed as a percentage of dry cell weight.

### Homology Modeling of IPMS

To analyze the effect of each amino acid substitution in IPMS, we constructed the wild-type and variant IPMS structures by homology modeling using SWISS-MODEL^2^. The template structure used for modeling was the structure of IPMS (LeuA) bound to L-leucine from *Mycobacterium tuberculosis* (PDB ID code: 3FIG). The amino acid sequence identity of the *M. tuberculosis* LeuA with IPMS from strain HC02-5-2 was 45.37%.

## Results

### Isolation and Characterization of Yeast Strain From Hibiscus Flowers for Awamori Brewing

We aimed to obtain a new yeast strain applicable to awamori brewing. Microorganisms were screened from wild hibiscus flowers in Okinawa for ethanol production. Among the isolated yeast colonies, strain HC02-5-2, with high-ethanol productivity, was further applied to genetic analysis in order to identify species. The BLAST search revealed that the 26S rDNA sequence of strain HC02-5-2 was identical to that of the yeast *S. cerevisiae.*

**FIGURE 1:**
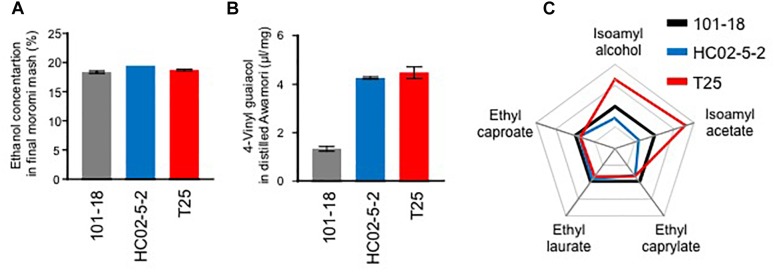
A laboratory-scale fermentation test of strains 101-18, HC02-5-2, and T25. **(A)** Ethanol concentrations in final fermented mash (*moromi*). **(B)** 4-Vinyl guaiacol concentrations (4-VG) in distilled *moromi*. The data are the mean, maximum, and minimum value of results from two independent experiments. **(C)** A radar chart of the initial volatile compounds. The each scale was standardized by the amounts derived from strain 101-18.

To examine the applicability of hibiscus yeast HC02-5-2 for awamori brewing, we performed a laboratory-scale awamori fermentation test. First, the concentration of ethanol in moromi fermented with HC02-5-2 reached 18.75%, whereas strain 101-18, a conventional awamori yeast, reached 17.71% (Figure 1A). This indicates that strain HC02-5-2 produces sufficient ethanol during fermentation.

### Comparative Analysis of Yeast Whole Genomes

**FIGURE 2:**
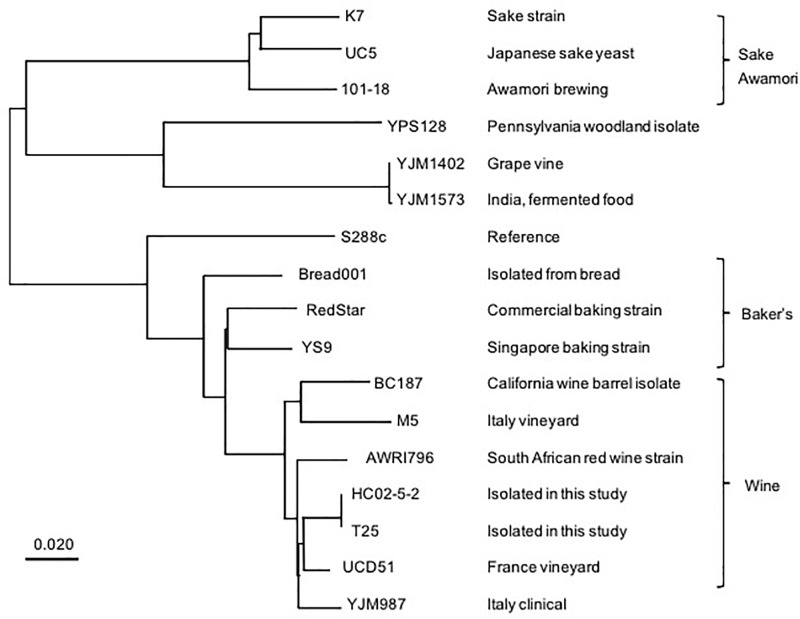
A phylogenic tree based on whole genome analysis of various *S. cerevisiae* strains. A single nucleotide variants-based phylogenic analysis was performed using the Neighbor-joining method. The BioSample database (https://www.ncbi.nlm.nih.gov/biosample/) information was used as a reference for the description of yeast strains. Scale bar represents 0.020 nucleotide changes per site.

We then conducted next-generation sequencing for the genome of strain HC02-5-2. As a result, 99.01% reads were mapped to the S288c reference genome and mean sequencing depth exhibited x113, confirming that strain HC02-5-2 belongs to *S. cerevisiae*. To examine the relationship between strain HC02-5-2 and other yeast strains used for fermentation, a phylogenic analysis using whole genome information was performed with comparing single-nucleotide variants (SNVs). Interestingly, the depicted phylogenic tree showed that strains HC02-5-2 and 101-18 were assigned to different clades of the tree. Strain HC02-5-2 is in a clade that includes yeast strains for wine brewing, whereas awamori strain 101-18 is in a clade that includes yeast strains for sake and shochu brewing (Figure 2). The evolutionary tree was also supported by phylogenic analysis focusing on specific genes, as shown in previous reports (Fay and Benavides, 2005; Futagami et al., 2017). These results suggest that strain HC02-5-2 does not share ancestry with sake or shochu yeast strains.

### Evaluation of the Flavor Compounds in Awamori Brewed by Hibiscus Yeast

The contents of odorants in awamori produced in a laboratory-scale test were measured. First, 4-VG was quantified by liquid chromatography. Its concentration observed in strain HC02-5-2 (6.47 μg/ml) was approximately 3 times higher than that observed in strain 101-18 (1.99 μg/ml) (Figure 1B). Based on a previous report (Mukai et al., 2010), the variants in the *PAD1* and *FDC1* genes essential for the decarboxylation of phenylacrylic acids were compared. It was confirmed that the intact and protein-coding *PAD1* and *FDC1* genes were present in the HC02-5-2 genome, whereas a nonsense mutant was found in the *FDC1* gene in strain 101-18. Next, the initial volatile compounds in awamori were quantified by gass chromatography. The concentration ratios of isoamyl alcohol and isoamyl acetate observed in strain HC02-5-2 were lower than those observed in strain 101-18 (Figure 1C). Although the initial volatile compounds in awamori brewed with strain HC02-5-2 were not prominent, the greater 4-VG production obtained with strain HC02-5-2 is desirable for awamori brewing.

### Isolation of Hibiscus Yeast Mutants With L-Leucine Accumulation From TFL-Resistant Mutants

We previously isolated TFL-resistant mutant strain 18-T55 with L-leucine accumulation from awamori yeast strain 101-18 (Takagi et al., 2015). By brewing with strain 18-T55, which overproduces isoamyl alcohol and isoamyl acetate, a new kind of awamori has been sucessfully commercialized. Therefore, to give distinctive characteristics to hibiscus yeast HC02-5-2, we attempted to isolate the TFL-resistant mutants that accumulate intracellular L-leucine. Strain HC02-5-2 was incubated in the presence of 6% EMS for 1 h and EMS-treated cells, which showed 30% of survival rate of untreated cells, were directly plated on SD agar medium containing 40 μg/ml of TFL. As a result, many TFL-resistant colonies were obtained, and among them one mutant strain, T25, exhibited larger amounts of L-leucine in the cells.

**FIGURE 3:**
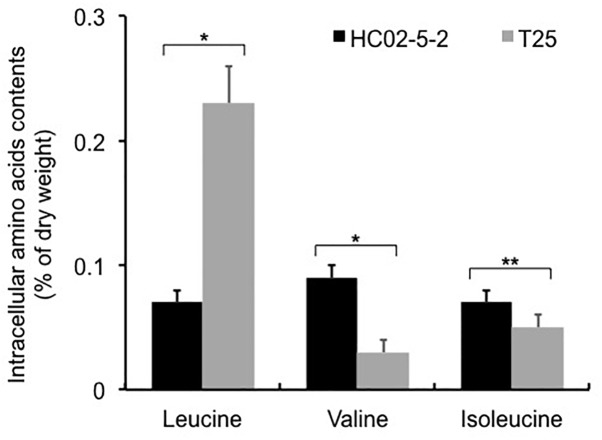
Intracellular amino acids contents in strains HC02-5-2 and T25. Yeast cells were cultured in 5 ml of SD+Am medium at 30°C for 2 days (OD_600_ = 10.0). Black and gray bars indicate strain HC02-5-2 and T25, respectively. The values are the means and standard deviations of results from three independent experiments. Statistically significant differences were determined by Student’s *t*-test (^∗^*p* < 0.01 and ^∗∗^*p* < 0.05).

Next, we determined the intracellular L-leucine quantities in both parent strain HC02-5-2 and mutant strain T25. As we expected, the intracellular concentration of L-leucine in strain T25 (0.225 ± 0.033% of dry cell weght) was significantly higher than that in parent strain HC02-5-2 (0.066 ± 0.006% of dry cell weght) (Figure 3). Interestingly, decreased concentrations of both L-valine and L-isoleucine were observed in strain T25 (Figure 3), suggesting that one or more mutations in strain T25 affect the biosynthetic pathway of branched-chain amino acids.

### IPMS Activity and the LEU4 Locus of a Hibiscus Yeast Mutant With L-Leucine Accumulation

**FIGURE 4:**
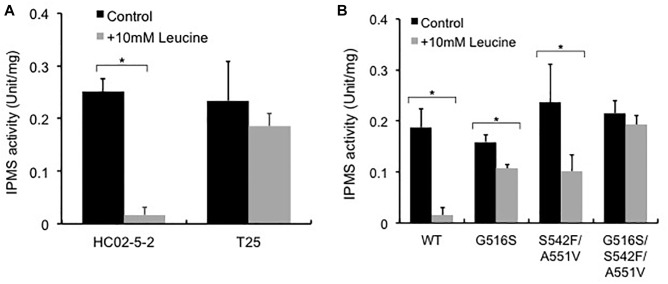
Effect of L-leucine on IPMS activity in **(A)** strains HC02-5-2 and T25, and **(B)** strain HC02-5-2 expressing the wild-type *LEU4* (WT), *LEU4*^G516S^ (G516S), *LEU4*^S542F/A551V^ (S542F/A551V), and *LEU4*^G516S/S542F/A551V^ (G516S/S542F/A551V). Yeast cell extract was used as enzyme solution. One unit of IPMS activity was defined as the amount of CoA generated from acetyl-CoA by the enzyme reaction at 37°C for 60 min. Black and gray bars indicate IPMS activities in the absence or presence of 10 mM L-leucine, respectively. The values are the means and standard deviations of results from three independent experiments. Statistically significant differences were determined by Student’s *t*-test (^∗^*p* < 0.01).

Previous reports indicated that reduced sensitivity to L-leucine feedback inhibition in the IPMS variants causes oversynthesis of L-leucine in the cell (Oba et al., 2005; Takagi et al., 2015). To elucidate the molecular mechanisms underlying L-leucine accumulation in strain T25, we examined IPMS activity in strains T25 and HC02-5-2. The IPMS activity in the presence or absence of 10 mM L-leucine was assayed using the crude cell extracts from both strains. As we expected, the IPMS activity from strain HC02-5-2 was remarkably inhibited by 10 mM L-leucine, indicating that the IPMS activity of this strain is regulated by L-leucine feedback inhibition. In contrast, the level of IPMS activity from strain T25 was approximately 80% even in the presence of 10 mM L-leucine, indicating that IPMS in mutant strain T25 is less sensitive to feedback inhibition by L-leucine than the parent strain HC02-5-2 (Figure 4A).

Next, we determined the DNA sequences of the *LEU4* gene coding IPMS in strains HC02-5-2 and T25. Gene amplification by PCR and the subsequent sequenncing result showed that strain T25 has a mixture of nucleotides A and G at position 1,546, whereas strain HC02-5-2 has a G at the same position. The mutation of G to A leads to the amino acid replacement of Gly to Ser at position 516, suggesting that strain T25 has a heteroallelic mutant of the *LEU4* gene.

### Effects of the LEU4 Mutations on IPMS Activity and L-Leucine Biosynthesis

To analyze the *LEU4* mutation identified in strain T25, the expression plasmids for the *LEU4* mutants were introduced to strain HC02-5-2. First, we assayed IPMS activity in the transformants overexpressing the *LEU4, LEU4*^G516S^, *LEU4*^S542F/A551V^, and *LEU4*^G516S/S542F/A551V^ genes. When the wild-type *LEU4* gene was overexpressed, IPMS activity was markedly inhibited in the presence of 10 mM L-leucine. As previously reported (Takagi et al., 2015), IPMS activity in cells overexpressing the *LEU4*^S542F/A551V^ gene was less sensitive than the wild-type *LEU4* gene to L-leucine. We also found that overexpression of the *LEU4*^G516S^ and *LEU4*^G516S/S542F/A551V^ genes increased IPMS activity in the presence of L-leucine (Figure 4B). These results indicated that the IPMS activity in strain T25 was mimicked by *LEU4*^G516S^ overexpression.

**FIGURE 5:**
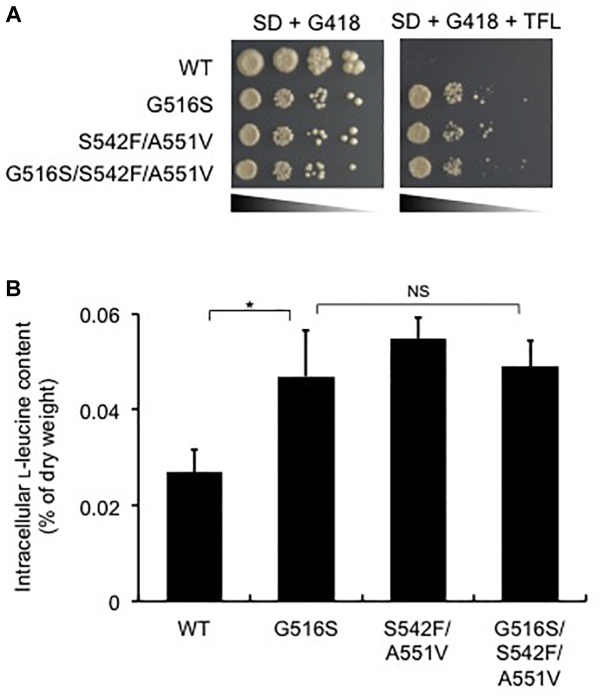
Effect of the *LEU4* mutation on L-leucine biosynthesis in various strains. **(A)** Growth phenotypes on SD+Alt medium containing 200 μg/ml G418 of strain HC02-5-2 expressing the wild-type *LEU4* (WT), *LEU4*^G516S^ (G516S), *LEU4*^S542F/A551V^ (S542F/A551V), and *LEU4*^G516S/S542F/A551V^ (G516S/S542F/A551V). After cultivation in SD+Am medium containing G418 at 30°C for 2 days, approximately 10^6^ cells of each strain and serial dilutions of 10^-1^ to 10^-4^ (from left to right) were spotted and incubated onto SD+Alt agar medium containing 200 μg/ml G418 plus 150 μg/ml TFL at 30°C for 2 days. **(B)** Intracellular L-leucine contents of strain HC02-5-2 expressing the wild-type *LEU4* (WT), *LEU4*^G516S^ (G516S), *LEU4*^S542F/A551V^ (S542F/A551V), and *LEU4*^G516S/S542F/A551V^ (G516S/S542F/A551V). Yeast cells were cultured in 5 ml of SD+Am medium at 30°C for 2 days (OD_600_ = 10.0). The values are the means and standard deviations of results from three independent experiments. Statistically significant differences were determined by Student’s *t*-test (^∗^*p* < 0.01). NS, not significant.

To check whether the *LEU4*^G516S^ gene is sufficient to confer TFL-resistance and L-leucine accumulation in strain HC02-5-2, we examined the growth of transformants on SD agar plates containing TFL and determined the intracellular L-leucine levels. Yeast cells overexpressing the wild-type *LEU4* gene could not grow in the presence of TFL (Figure 5A). On the other hand, overexpression of the *LEU4*^G516S^, *LEU4*^S542F/A551V^, and *LEU4*^G516S/S542F/A551V^ genes resulted in resistance to TFL. In these transformants, the intracellular L-leucine concentration was higher than that in wild-type strain HC02-5-2 (Figure 5B). These results indicate that the Gly516Ser variant of IPMS reduces sensitivity to L-leucine, leading to L-leucine accumulation in the hibuscus yeast strain.

### Awamori Fermentation Test for T25 and Analysis of Ethannol and Flavor Compounds

**FIGURE 6:**
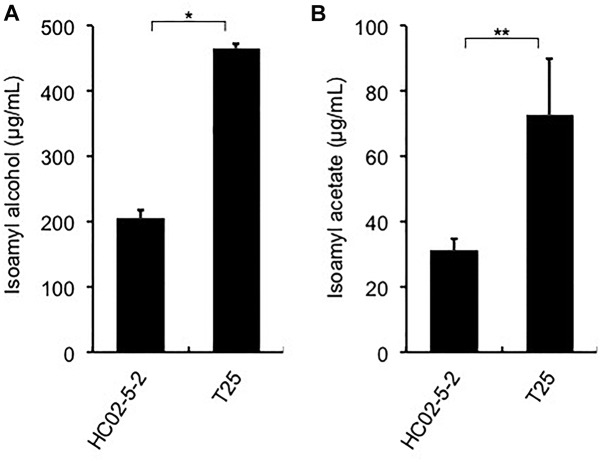
**(A)** Isoamyl alcohol and **(B)** isoamyl acetate concentrations in distilled *moromi* prepared from strains HC02-5-2 and T25 by a laboratory-scale fermentation test. The values are the means and standard deviations of results from three independent experiments. Statistically significant differences were determined by Student’s *t*-test (^∗^*p* < 0.01 and ^∗∗^*p* < 0.05).

Finally, we evaluated the potential of strain T25 for awamori brewing. First, to determine ethanol productivity, a laboratory-scale fermentation test was carried out. The concentration of ethanol in final moromi fermented with strain T25 reached 18.05%, which is equivalent to that with strains HC02-5-2 and 101-18 (Figure 1A). Next, we analyzed distilled awamori by gas chromatography. As we expected, in proportion to the cellular L-leucine level, strain T25 produced 2.3-fold more isoamyl alcohol and isoamyl acetate than strain HC02-5-2 (Figure 6). On the other hand, the concentrations of ethyl caprylate, ethyl laurate, and ethyl caproate were almost the same among the 3 strains (HC02-5-2, T25, and 101-18) (Figure 1C), suggesting that breeding selectively promotes the production of odorant compounds in awamori. Thus, mutan strain T25 derived from hibiscus yeast is expected to be applicable to awamori brewing.

## Discussion

By virtue of its high productivity of ethanol and favorable flavors, strain 101-18, an awamori yeast strain of *S. cerevisiae*, has been used commercially to brew awamori by most awamori manufacturers (Shinzato et al., 1989). Since demands to expand the diversity of awamori qualities have increased, the development of novel yeast strains that vary in taste and flavor may contribute to the awamori industry. In this study, we found that novel yeast strain HC02-5-2, isolated from hibiscus flowers, produced enough ethanol for awamori brewing. In fact, “Hibiscus Awamori”, a new kind of awamori brewed with strain HC02-5-2, has been sold on the Japanese market. Subsequent breeding of strain HC02-5-2 succeeded in obtaining the desirable mutant T25, which produces more isoamyl alcohol and isoamyl acetate than HC02-5-2, which imbue awamori with fruity flavors. In a pilot-scale fermentation, greater amounts of isoamyl alcohol and isoamyl acetate were produced in awamori fermented by strain T25 than in those produced by strain 101-18 (data not shown). Our data indicate that strains HC02-5-2 and T25 are suitable for awamori brewing.

Strain HC02-5-2 produced more 4-VG producion than strain 101-18 (Figure 1B). For several alcoholic beverages, such as beer, 4-VG is recognized as phenolic off flavor (POF). A comparative study revealed that the yeast strains with strong domestication like beer strains exhibit the POF negative phenotype (Gallone et al., 2016). In awamori, 4-VG is known as a precursor compound of vanillin, which confers sweet vanilla flavor and is characteristic of aged awamori (Maeda et al., 2018). During storage, awamori flavors are commonly believed to change, and storage methods for beneficial aging have been established (Kanauchi, 2012). One of the molecular bases for awamori aging is explained by the conversion of 4-VG into vanillin (Koseki et al., 1996). Therefore, strain HC02-5-2 is suggested to have a distinct feature for awamori brewing, whereas strain 101-18 is a POF-negative strain with a nonsense mutation in the *FDC1* gene.

The initial scented fruity flavors are preferable to awamori and sake consumers. Several studies have increased fruity aromatic compounds in Japanese sake by introducing spontaneous one or mor mutations into industrial yeast strains (Arikawa et al., 2000; Takahashi et al., 2017). We recently reported that L-leucine analog-resistant mutants overproduced isoamyl alcohol and isoamyl acetate (Takagi et al., 2015). Since strain HC02-5-2 showed no striking features in initial volatile compounds, we expected to breed this yeast strain for a diversity of tastes and flavors. As a result, the obtained mutant strain T25 with L-leucine accumulation produced more isoamyl alcohol and isoamyl acetate than its parent strain HC02-5-2. It is known that an increase in L-leucine leads to high-levels of isoamyl acetate production (Ashida et al., 1987; Quilter et al., 2003; Takagi et al., 2015). Moreover, strain T25 produced mor isoamyl acetate than strain HC02-5-2 did. Isoamyl acetate is converted mainly from isoamyl alcohol catalyzed by alcohol acetyltransferase (the *ATF1* gene product) in *S. cerevisiae* (Inoue et al., 1997). In the present study, we did not observe any sequence difference in the *ATF1* gene between strains HC02-5-2 and T25. Further investigation is needed to understand the mechanism by which strain T25 overproduces isoamyl acetate.

Interestingly, strain T25 produced approximately 3 times more L-leucine than the parent strain, whereas intracellular levels of L-valine and L-isoleucine in strain T25 were decreased to about 30 and 70% of those observed in the parent strain, respectively. Previous studies have shown that the balance of intracellular amino acids is tightly controlled in *S. cerevisiae* (Watson, 1976; Messenguy et al., 1980). Moreover, L-valine and L-isoleucine, which are categorized as branched-chain amino acids (BCAAs) containing L-leucine are bioynthesized by commonly sharing the same enzymes (acetolactate synthase, acetohydroxiacid reductoisomerase, and dihydroxiacid dehydratase) in the first four steps in mitochondria (Kohlhaw, 2003). It is possible that an increase in L-leucine accounts for a decrease in other BCAAs in yeast cells. We found that L-leucine accumulation in strain T25 is caused by expression of the Gly516Ser variant of IPMS (Figure 3, 5B). IPMS is the key enzyme that regulates L-leucine biosynthesis via the mechanism of negative feedback inhibition by L-leucine in *S. cerevisiae*. Homology modeling analysis suggests that Gly516 is located on α 14 helix comprising the L-leucine binding site, which is the allosteric regulation domain with L-leucine in the bacterial IPMS (Koon et al., 2004). Furthermore, the amino acid change of Gly to Ser at position 516 in IPMS was supposed to directly interfere with L-leucine binding due to the steric hindrance at the binding cleft, leading to the desensitization of the L-leucine feedback inhibition of IPMS (Supplementary Figure 1).

## Conclusion

In conclusion, newly isolated strain HC02-5-2 from hibiscus flowers was identified as a *S. cerevisiae* strain related to the wine lineage of this species. Strains HC02-5-2 and its mutant T25 possess favorable characteristics for developing both the initial scented fruity flovors and the sweet flavors associated with aging. Our data supported the practical use of these isolated yeast strains. Moreover, we can now explore the certain molecular basis of fermentation properties of these strains. Since spirits contain many fragrant ingredients and their balance detrmines the quality, it is important to control multiple flavor compounds. This combinatorial approach to yeast isolation from nature and its breeding is applicable to the variation of the quality of alcoholic beverages in the fermentation industry.

## Author Contributions

DW, MT, and HT conceived the study and designed the experiments. TA, YT, YS, HA, and KT performed the experiments. TA, YT, RN, DW, MT, and HT analyzed the data. TA, YT, and HT wrote the manuscript. All authors reviewed and approved the final version of manuscript.

## Conflict of Interest Statement

TA, HA, KT, and MT were employed by BioJet Co. The remaining authors declare that the research was conducted in the absence of any commercial or financial relationships that could be construed as a potential conflict of interest.

## Footnotes

1 https://github.com/BiojetCoLtd/GW_to_phylogeny

2 http://swissmodel.expasy.org/
