# Supplementary material for: Characterization of a New Saccharomyces cerevisiae Isolated From Hibiscus Flower and Its Mutant With L-Leucine Accumulation for Awamori Brewing
Source: Front Genet. 2019 May 28;10:490. doi: 10.3389/fgene.2019.00490 (PMC6558412; doi:10.3389/fgene.2019.00490)

**Supplemental Figure 1** Schematic diagrams of the predicted l-leucine-binding site in IPMSs of strains HB and T25. Conformational state of wild-type (HC02-5-2) and variant (T25) IPMSs bound with l-leucine was referred to the structure of the *Mycobacterium tuberculosis* IPMS (LeuA) (PDB ID code: 3FIG). A substituted residue in IPMS (Gly to Ser at posituion 516) and l-leucine located in the binding site are shown in stick model. l-Leucine suggested to be combined apparently in the binging site of wild-type IPMS. However, Ser516 in variant IPMS was considerred to interfere l-leucine binding.

**Supplemental Table 1** Sequence data used in the phylogenic analysis


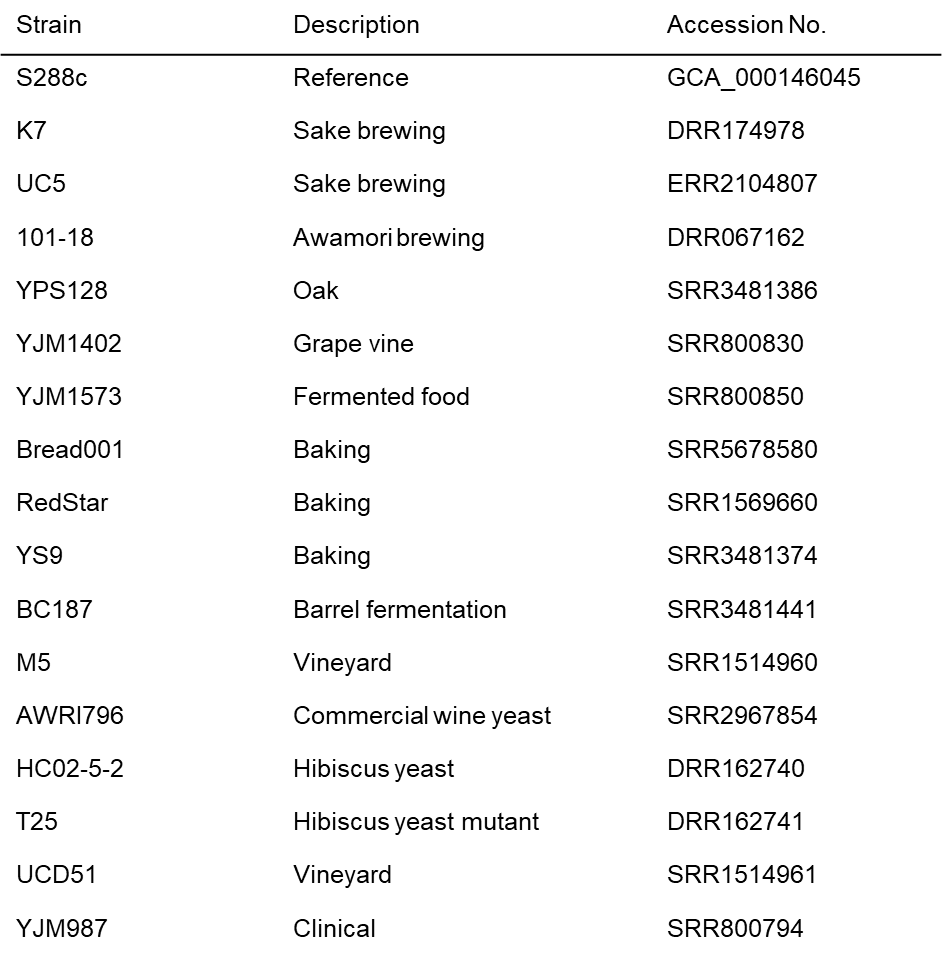

Supplement: Supplementary file 1 [file Table_1.DOC]
